# Optimizing the measurement of sample entropy in resting-state fMRI data

**DOI:** 10.3389/fneur.2024.1331365

**Published:** 2024-02-15

**Authors:** Donovan J. Roediger, Jessica Butts, Chloe Falke, Mark B. Fiecas, Bonnie Klimes-Dougan, Bryon A. Mueller, Kathryn R. Cullen

**Affiliations:** ^1^Department of Psychiatry and Behavioral Sciences, Medical School, University of Minnesota (UMN), Minneapolis, MN, United States; ^2^Division of Biostatistics and Health Data Science, School of Public Health, UMN, Minneapolis, MN, United States; ^3^Psychology Department, College of Liberal Arts, UMN, Minneapolis, MN, United States

**Keywords:** sample entropy (SampEn), fMRI, R software, brain dynamics, complexity

## Abstract

**Introduction:**

The complexity of brain signals may hold clues to understand brain-based disorders. Sample entropy, an index that captures the predictability of a signal, is a promising tool to measure signal complexity. However, measurement of sample entropy from fMRI signals has its challenges, and numerous questions regarding preprocessing and parameter selection require research to advance the potential impact of this method. For one example, entropy may be highly sensitive to the effects of motion, yet standard approaches to addressing motion (e.g., scrubbing) may be unsuitable for entropy measurement. For another, the parameters used to calculate entropy need to be defined by the properties of data being analyzed, an issue that has frequently been ignored in fMRI research. The current work sought to rigorously address these issues and to create methods that could be used to advance this field.

**Methods:**

We developed and tested a novel windowing approach to select and concatenate (ignoring connecting volumes) low-motion windows in fMRI data to reduce the impact of motion on sample entropy estimates. We created utilities (implementing autoregressive models and a grid search function) to facilitate selection of the matching length *m* parameter and the error tolerance *r* parameter. We developed an approach to apply these methods at every grayordinate of the brain, creating a whole-brain dense entropy map. These methods and tools have been integrated into a publicly available R package (“powseR”). We demonstrate these methods using data from the ABCD study. After applying the windowing procedure to allow sample entropy calculation on the lowest-motion windows from runs 1 and 2 (combined) and those from runs 3 and 4 (combined), we identified the optimal *m* and *r* parameters for these data. To confirm the impact of the windowing procedure, we compared entropy values and their relationship with motion when entropy was calculated using the full set of data vs. those calculated using the windowing procedure. We then assessed reproducibility of sample entropy calculations using the windowed procedure by calculating the intraclass correlation between the earlier and later entropy measurements at every grayordinate.

**Results:**

When applying these optimized methods to the ABCD data (from the subset of individuals who had enough windows of continuous “usable” volumes), we found that the novel windowing procedure successfully mitigated the large inverse correlation between entropy values and head motion seen when using a standard approach. Furthermore, using the windowed approach, entropy values calculated early in the scan (runs 1 and 2) are largely reproducible when measured later in the scan (runs 3 and 4), although there is some regional variability in reproducibility.

**Discussion:**

We developed an optimized approach to measuring sample entropy that addresses concerns about motion and that can be applied across datasets through user-identified adaptations that allow the method to be tailored to the dataset at hand. We offer preliminary results regarding reproducibility. We also include recommendations for fMRI data acquisition to optimize sample entropy measurement and considerations for the field.

## Introduction

The complexity of brain signals contains information that may have critical importance for neuroscience and psychiatry. Broadly, it has been recognized across physiological systems that more complex (e.g., irregular) dynamic patterns allow the organism to better adapt to unexpected stressors (1–3). A concept born in physics but more recently adopted to information theory, entropy is a measure of unpredictability of a signal, a measure that indicates complexity. Entropy can be calculated from time series such as those from signals measured during resting-state functional magnetic resonance imaging (fMRI) to quantify the unpredictability and complexity of a brain state.

**Figure 1:**
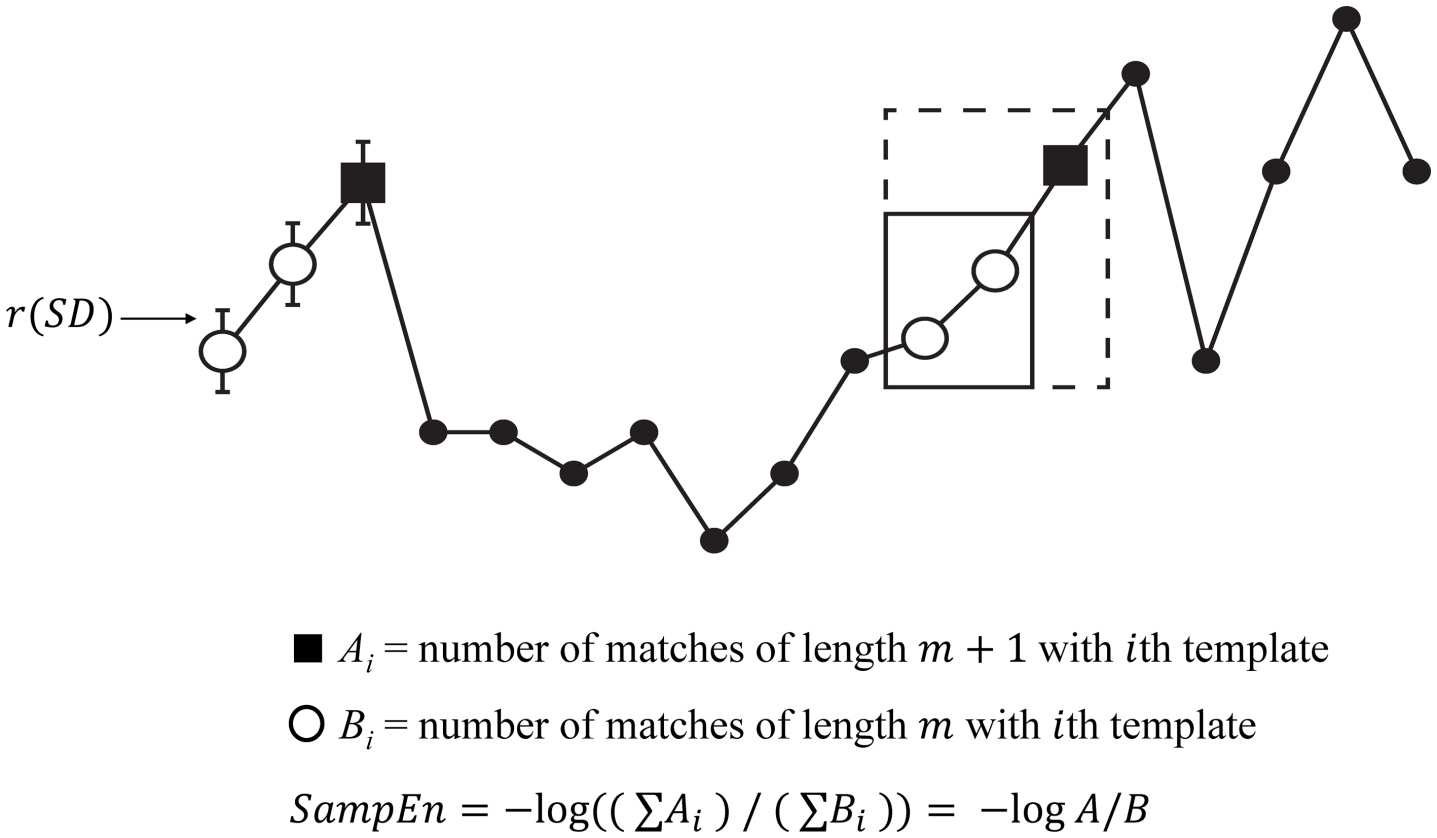
Overview of sample entropy. This demonstration of entropy estimation using SampEn was adapted with permission from Richman et al. (16; page 10). In this example, *m* is 2 and the threshold for accepting a match is *r* multiplied by the standard deviation (see error bars). Note that the template (first two points) is matched by the 11th and 12th points (solid box), and that the *m* + 1st points also match (dashed box). In this case, quantities of *A* and *B* both increase by 1.

There are many different ways of measuring entropy in brain signals, but many of the classical entropy calculations are not appropriate for the relatively short and noisy time series derived from physiological measurements (4). One of the first entropy measures suited for such a purpose was Approximate Entropy (5). Richman and Moorman (4) extended this work to develop sample entropy (SampEn) to overcome two main limitations of approximate entropy: (1) the strong relationship between approximate entropy and the length of the time series and (2) the lack of relative consistency. As shown in Figure 1, SampEn compares segments of the time series to a template of length *m* + 1. If the first *m* timepoints match the template (within a tolerance factor *r*) the segment is listed as an “*m* match.” If all *m* + 1 timepoints match the template within the tolerance then the segment is also listed as an “*m* + 1 match.” The template-matching process is repeated so that each segment is considered a template once, and is also assessed for matching the other segments many times. The proportion of *m + 1* matches to *m* matches is considered a measure of complexity (i.e., if a high proportion of the length *m* matches are also length *m* + 1 matches, then the time series is predictable and has low complexity). SampEn is the negative log of this proportion. As described by Lake et al. (6), higher *m* values and lower *r* values tend to reduce both the number of length *m* matches (Cm) and the number of length *m* + 1 matches (Cm1). The main difference between approximate entropy and SampEn is that approximate entropy will count self-matches whereas SampEn does not. In other words, the template is compared to itself in approximate entropy, whereas the template is not compared to itself in SampEn. The counting of self-matches in approximate entropy leads to bias resulting in the drawbacks addressed by SampEn. However, not counting self-matches could result in either Cm or Cm1 equaling 0; if this happens, SampEn is not mathematically defined.

Richman and Moorman (4) applied SampEn to cardiac R-R intervals measured on infants (these time series consisted of 4,096 measurements approximately 0.3 s apart), but these methods can also be applied to calculate entropy from time series such as those from signals measured during resting-state functional magnetic resonance imaging (fMRI) or electroencephalography (EEG) to quantify the unpredictability and complexity of a brain state. Much of the brain entropy literature has utilized data from electroencephalography (EEG), which has the advantage of very dense temporal sampling and is more similar to the cardiac signals used by Richman and Moorman. The utilization of fMRI data offers the advantage of examining deeper regions of the brain, such as the limbic regions implicated in depression (7, 8), but also presents additional challenges that need to be addressed.

First, the extent to which motion may impact SampEn measurement has been largely ignored in the fMRI literature. However, some research has suggested that entropy measurements (including SampEn) may be highly sensitive to the effects of motion (9), underscoring this as an area in need of attention. In calculating SampEn, motion events can substantially distort the shape of the time series, leading to erroneous assessments of its irregularity in that segment. Since the standard deviation of the timeseries is factored into the calculation of the tolerance threshold *r,* motion events can inflate the number of “matches” such that fluctuation patterns that in actuality are irregular are deemed as similar only because the tolerance *r* is erroneously high. Head motion during scanning is well known to influence resting-state fMRI data, and methods have been developed to optimize calculations of functional connectivity to minimize the impact of head motion on the data. Such methods have included scrubbing or de-weighting volumes that have high motion or replacing them with interpolated data (10–13). However, scrubbing results in problematic discontinuities in the data and procedures involving interpolation may distort the SampEn measurement. Dong et al. (14) introduced a modified version of SampEn to account for missingness in a time series and compared their approach to other existing methods for dealing with non-continuous time series. Their proposed method resulted in lower percent errors when missingness was introduced into example data compared to other methods. Quantifying and understanding how to best minimize motion-related confounds for calculating SampEn with resting-state fMRI data is needed.

Second, calculation of SampEn requires the selection of parameters for template length *m* and error tolerance *r*. A prior resting-state fMRI study tested a range of parameters to identify those which minimized the relative error of SampEn that was calculated on time series from cerebrospinal fluids, and then applied these parameters to calculate SampEn on time series from gray matter regions. Their data suggested accuracy of SampEn estimates was maintained by a tradeoff between pattern length *m* and tolerance factor *r* (15). As recommended by the creators of SampEn (4, 16), these parameters should be selected based on the input data. However, more common practice has been for fMRI researchers to select the parameters based on past publications (17). The field would benefit from tools that could allow these parameters to be automatically selected based on the input data.

In an effort to address the above concerns, we developed the Parameter Optimization and Windowing for Sample Entropy in R (“powseR”) toolkit for calculating SampEn entropy in brain data. PowseR is an R package that includes functions for performing bandpass filtering (with or without the interpolation of high motion timepoints), optimizing SampEn parameter selection (m and r), and calculating entropy at the grayordinate (dense time series or dtseries) or parcel (parcellated time series or ptseries) level in a CIFTI image file using a novel windowing method to exclude motion-corrupted volumes. PowseR will be made available to the general public via Github and was developed using R v4.1.0 (18).

We applied powseR to baseline resting-state fMRI data from children (9–10 years old) in the Adolescent Brain and Cognitive Development (ABCD) Study (19). We conducted initial testing to confirm the impact of the windowing approach on the entropy values and their relationship to motion, using both the optimal parameters identified by powseR based on the data, and using other commonly-used parameters. We applied the methods we had developed to calculate SampEn at every cortical vertex and every subcortical voxel in the brain (two measurements per person from two different parts of their baseline scanning session) and then conducted statistical testing on these data to evaluate reliability across repeated scans as well as sensitivity to head motion.

## Materials and methods

### Overview of powseR functionality

PowseR is an R package that facilitates SampEn calculation in modern, grayordinate-space neuroimaging data. Beyond the SampEn calculation itself, powseR contains several functions to help users avoid motion-driven distortions of SampEn values and select optimal parameters (*m* and *r*) for their data. This section describes the various features available in powseR.

#### Windowing approach to reduce bias from head motion

The windowing procedure in powseR was developed with the goal of extracting the best parts of the data (those least influenced by motion) prior to calculating SampEn while preserving as much naturally-continuous data as possible. We adopted a procedure introduced by Dong et al. (14) to handle missing values in time series data from physiological signals, which added a screening condition that required nonmissing values in the template matching process (14). Their approach had the advantage of avoiding any modification of the input data, leaving the natural structure of the data intact. However, while SampEn is relatively stable at varying record lengths, this approach may introduce bias by censoring differing numbers of timepoints from each observation (14, 16, 20). To address this, our application of this method adds a novel windowing procedure that extracts a predetermined number of fixed-length windows consisting of continuous time points (volumes) that meet a user-defined framewise displacement (FD) threshold.

Given a user-defined FD threshold, window length (number of volumes) and desired number of windows, powseR’s windowing algorithm searches the time series for low motion windows using a flexible approach that does not rely on fixed indices. For example, in a series of 60 volumes with high motion events at volumes 10 and 55, a fixed indices-based windowing procedure might consider three 20-volume windows (volumes 1–20, 21–40, and 41–60) and only find the second window to be usable. PowseR’s procedure would define two usable windows out of the data, e.g., volumes 11–31 and 32–52. In the event that the number of candidate low-motion windows found exceeds the number requested by the user, windows with the highest mean FD are discarded.

**Figure 2:**
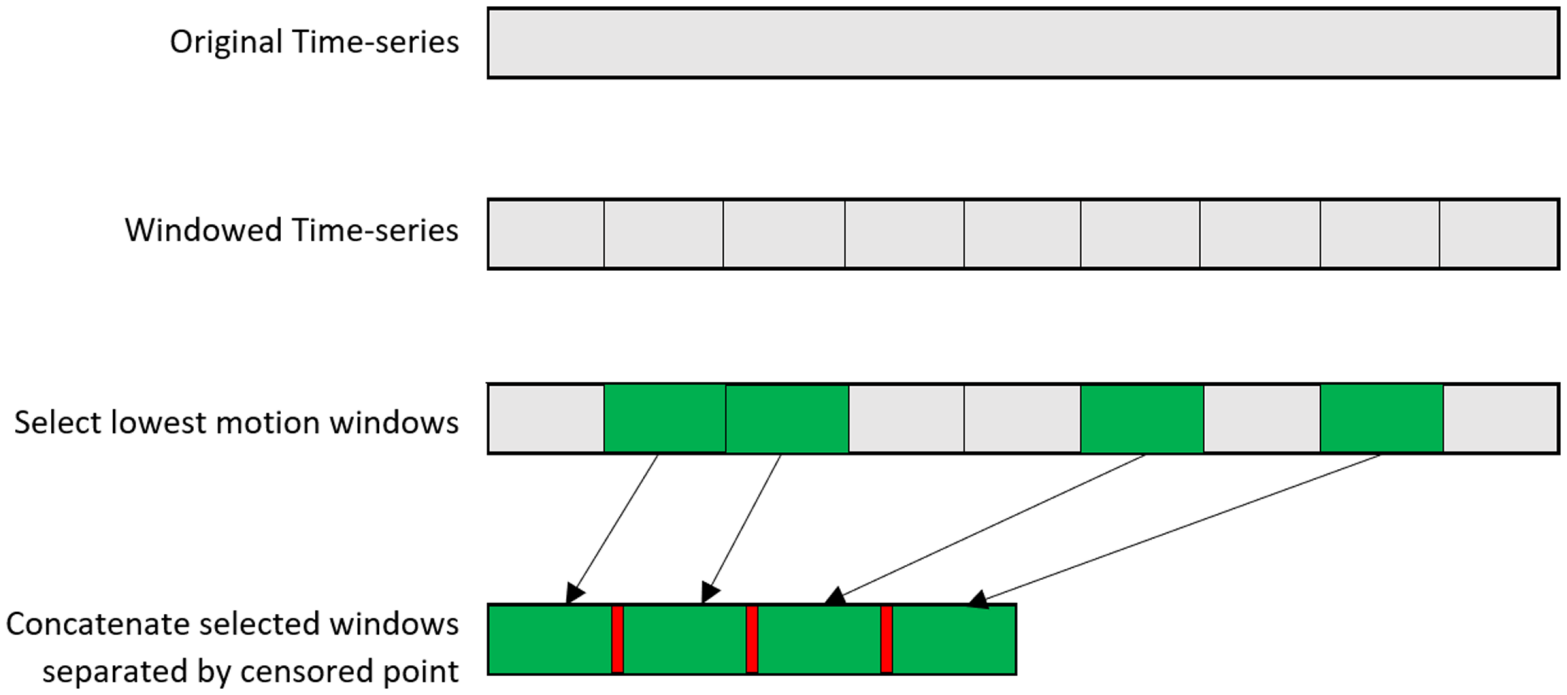
Schematic of the windowing procedure. Low-motion windows are selected and concatenated, with an ignored volume in between windows.

This approach is intended to minimize the influence of large head motion on the final entropy estimate while also harmonizing the length and number of censored data points across observations. The selected windows are concatenated with a censored time-point between each as shown in Figure 2. A censored time point is included between all windows even if they were adjoining each other in the original time series, so that all time series have the same number of censored time points. SampEn is then calculated across the new time series using the method provided in Dong et al. (14) to ignore censored time points. Alternatively, the user may opt to forgo the windowing approach and instead consider the full, uninterrupted time series when calculating SampEn.

#### Temporal filtering with interpolation

**Figure 3:**
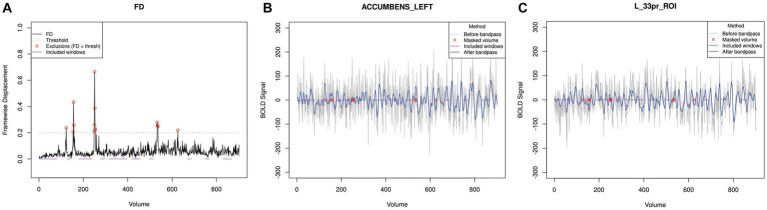
Time series processing for a single subject. Panel **(A)** illustrates the variation in Framewise Displacement for a single participant over the course of the scan. Red circles indicate the volumes where FD values exceeded the acceptable threshold of 0.2 mm. These windows were masked out prior to application of the bandpass, and were never included in the “best” windows that were selected for analysis (indicated with purple lines). Panels **(B)** and **(C)** illustrate BOLD signal patterns from a single subject in the left nucleus accumbens **(B)** and left anterior cingulate cortex **(C)**. The gray line shows the raw BOLD signal time series; the blue line shows the time series after applying the bandpass filter. Red x marks indicate volumes that were masked out prior to bandpass application because of excessive motion (FD > 0.2 mm).

In addition to motion, noise (either physiological or equipment-generated) may artificially inflate entropy measurements by contributing variability that is not neural in origin. A common method of isolating signals of neural origin is to apply a bandpass filter. However, applying a bandpass filter to data that includes large motion events causes motion-related spikes to propagate into low motion volumes (21). To address this, powseR includes an option to interpolate high-motion volumes prior to filtering. Because these high-motion volumes are excluded when using the windowing approach, SampEn calculations will never include volumes that had been interpolated. This process is demonstrated in Figure 3 which was generated using a function available in powseR.

#### Parameter (*m* and *r*) optimization

While powseR allows for the values of *m* and *r* to be manually defined by the user, two functions are included to provide data-informed suggestions for optimal parameters. In the *select_m* function, we follow the methods of (6, 16) to fit an autoregressive model to the data, using the optimal order (*m*) of the model based on the AIC to set the matching length *m* parameter. Because the AR model used in *select_m* does not account for the concept of the *r* parameter, the next step is to find an appropriate *r* value for the suggested *m*. A second function, *search_mr_grid*, is used to select the error tolerance *r* parameter using a criterion based on $\sigma_{cp}^{2}$ which minimizes the maximum of the relative error of the SampEn estimate and CP (=Cm1/Cm) estimate. The *search_mr_grid* tool is based on a process described in (6), where after determining m, several potential *r* values are explored to find an *r* value such that the number of matches (Cm) is not too small and the conditional probability (CP = Cm1/Cm) is not too close to 1. Multiple candidate *m* values may be included when running *search_mr_grid* to search for optimal *m* and *r* values simultaneously. However, an important aspect to consider in this decision is the computational demand (despite powseR’s support for multiprocessing) of considering many possible combinations of parameters, so we suggest that users first choose *m* by using the much less computationally intensive AR approach (*select_m*) and then use *search_mr_grid* to assess various values of *r* in combination with the single chosen *m* or, if desired, a small range of candidate *m* values (i.e., the suggested *m* +/− 1).

#### Calculating sample entropy

Following parameter selection, the *calc_entropy* function in powseR performs the final SampEn calculation, including options to apply the temporal filtering and windowing procedures described previously. Leveraging the *cifti* (22) and *ciftiTools* (23) packages in R, this calculation is performed at each grayordinate (surface vertex or subcortical voxel) in a dtseries CIFTI file or each parcel in a ptseries file. The resulting entropy values can also be written out as CIFTI files, allowing the subsequent use of existing tools for visualizing and analyzing CIFTI data, such as Connectome Workbench (24) and Permutation Analysis of Linear Models (25).

### Applying powseR to the ABCD dataset to assess reliability and sensitivity to head motion

We applied the methods described above to the resting-state data fMRI collected at baseline in the ABCD study. The ABCD Study is a longitudinal study of over 11,000 children living in the United States who were 9–10 years old at study entry and demographically matched to the United States population. The fMRI acquisition includes 20 min of resting-state data collected across four runs. The ABCD data is publicly available through the National Data Archive.^1^

Prior to analysis, we applied some customizations to the publicly available data that we expected would optimize the data for our analysis. Specifically, we reran the final step of the ABCD-HCP pipeline with a broader bandpass filter (0.009 and 0.25 Hz) in order to preserve higher frequency fluctuations that may be of interest (26, 27) and included zero-padding at the beginning and ends of the scan prior to filtering to correct a known “edge effect” artifact in the publicly available data. This temporal filtering was performed within the ABCD-HCP pipeline (no filtering was performed by powseR). Notably, the ABCD-HCP pipeline includes an interpolation step before applying the bandpass that is equivalent to powseR’s implementation. For this analysis, we used baseline visit resting state images from 3,058 subjects who had 20 windows meeting our criteria (20 continuous data points with FD < 0.3 mm) in BOTH the earlier segment (runs 1 + 2), and the later segment (runs 3 + 4). We utilized the windowing procedure to select the 20 best windows across runs 1 and 2, and separately, the 20 best windows across runs 3 and 4. (The first 10 volumes of each run were always excluded from consideration.) Thus we had two SampEn measures for each person, one taken early in the scanning session, and one taken later on in the same scanning session (there was typically a gap of approximately 20 min in between rest run 2 and run 3, during which other types of neuroimaging data was collected). After the windowing procedure, the *select_m* tool was applied to identify the optimal value for *m* followed by the *search_mr_grid* tool to determine the best *r* across all brain regions and across all subjects. Finally, we used these optimized parameters to calculate SampEn at every grayordinate for the 3,058 study participants for both the early (run 1 and 2) and later (run 3 and 4) datasets. Group mean entropy maps averaged across the 3,058 sessions were computed for the earlier and later resting state pairs. The results were inspected to appreciate the distribution of entropy values across the brain.

#### Assessing the performance of the windowing method in reducing sensitivity to head motion

To assess whether powseR’s windowing function mitigates the impact of head motion on entropy values, we correlated mean SampEn values (across grayordinates) with mean FD (across runs 1 and 2). To compare our windowing method with a more conventional approach, we then repeated these steps on the same set of participants but using a SampEn calculation that was based on the full, uninterrupted time series (except the initial 10 volumes). For this conventional approach, SampEn values were calculated separately for runs 1 and 2 and averaged across runs. The resulting mean SampEn values were then correlated with mean FD (across runs 1 and 2).

#### Assessing reliability across repeated measurements

Grayordinate-wise intraclass correlation coefficients (ICC) were used to assess reliability of the SampEn measurement across the brain. We calculated the ICC by first fitting the following random effects ANOVA model: μij = μ + αi + eij, where μij is the j-th entropy measurement from person i, αi is a random intercept to capture the variation across individuals, and eij is noise. From here, the ICC can be calculated as the ratio of the variance attributed to the individuals, as measured by the variance of the random intercept, to the total variability in the measurements, as measured by the sum of the variance of the random intercept and noise. This is an approach previously used in the neuroimaging literature [e.g., (28, 29)]. For estimating the variance components of this model, we used the lme4 package in R.

## Results

### Characterizing whole-brain dense entropy in the ABCD study data

**Figure 4:**
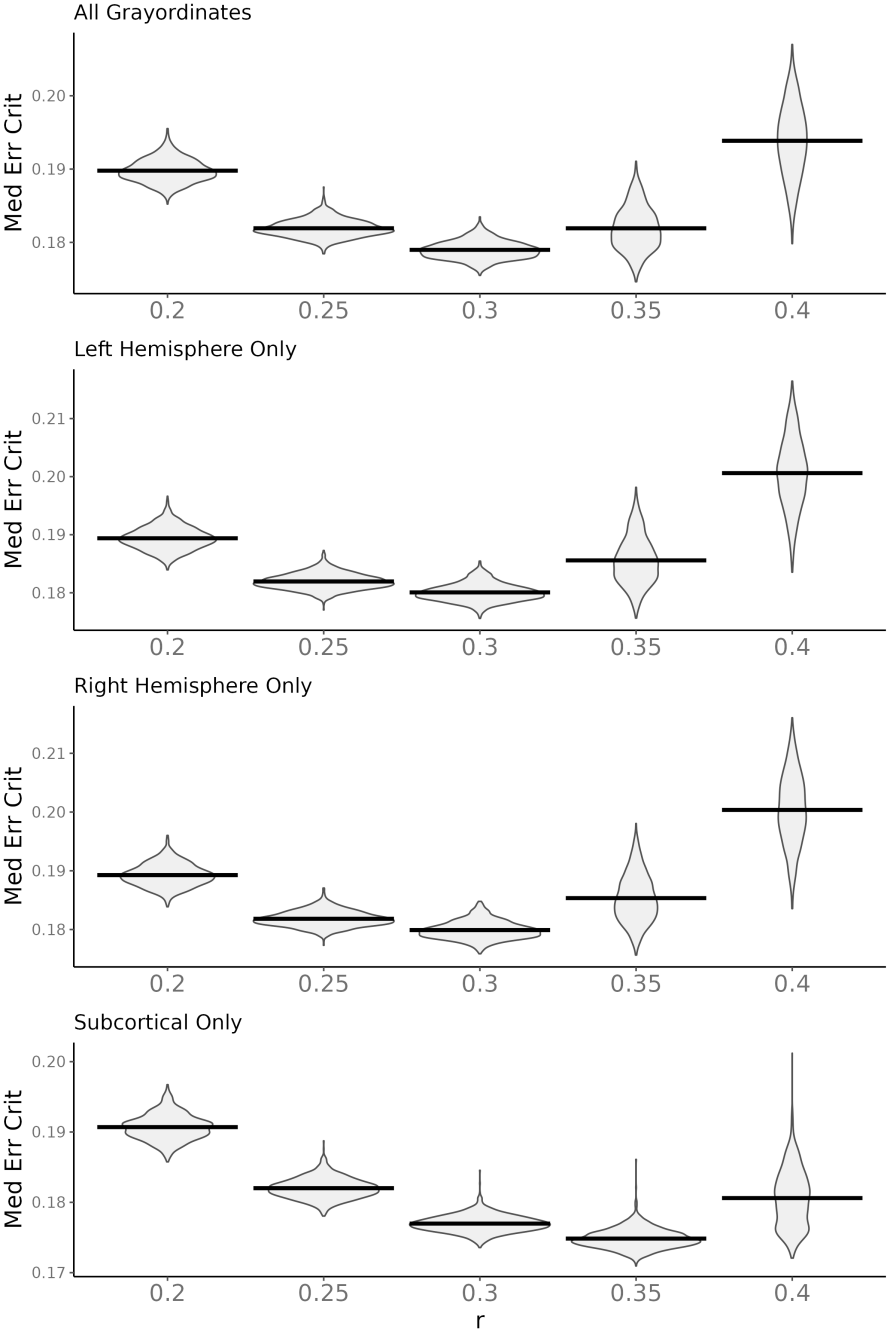
Results from *search_mr_grid*. Violin plots showing the distribution of median error criterion for *m* = 2 and various *r* values across subjects. The top panel shows median error criterion across all grayordinates. The lower three panels show each of the cortical hemispheres and the subcortex separately.

**Figure 5:**
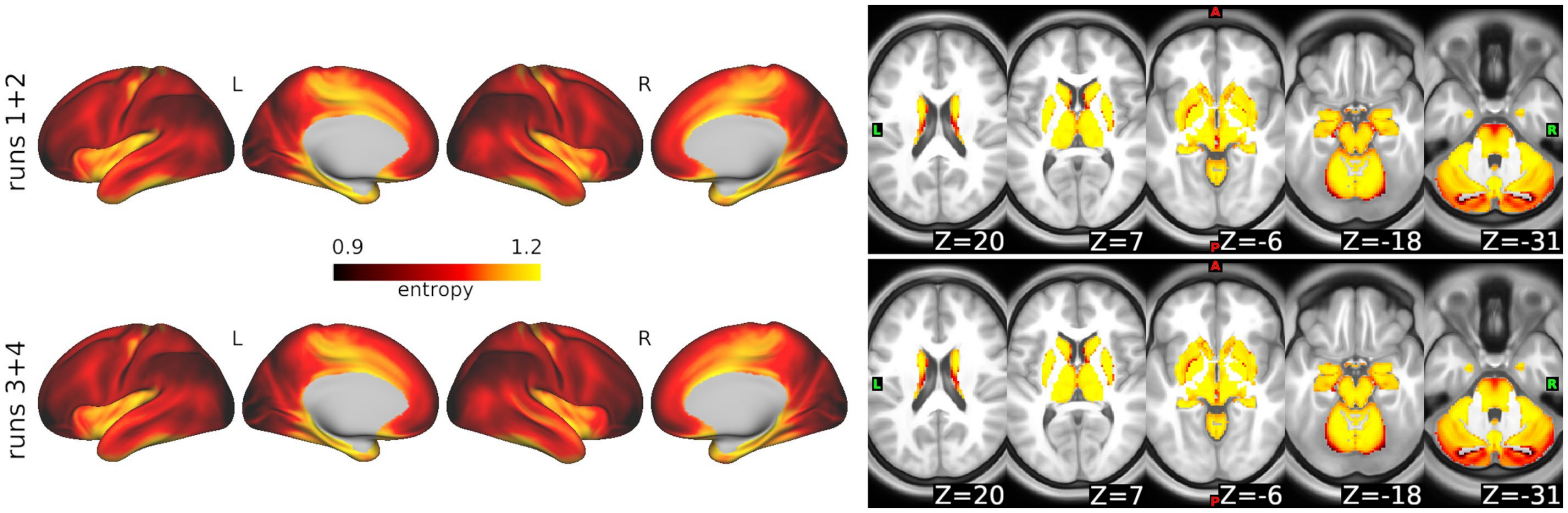
Whole-brain sample entropy in the ABCD Study data. Top: Average sample entropy across 3,058 participants for runs 1 + 2. Bottom: Average for runs 3 + 4.

In the ABCD data, *select_m* identified *m* = 2 as optimal using the windowed approach. We then used *search_mr_grid* to consider several *r* values for *m* = 2. Figure 4 shows the resulting median error criterion maps, which suggested that for cortical regions, the optimal *r* = 0.3, while for subcortical regions, the optimal *r* = 0.35. For the purposes of simplicity, we utilized *m* = 2 and *r* = 0.3 to calculate SampEn at every grayordinate. Notably, these parameters have been frequently used for fMRI in prior literature (30–32). Figure 5 shows group mean maps for runs 1 + 2 and runs 3 + 4. As shown, entropy values are higher in medial cortical regions including dorsal cingulate cortex, supplementary motor area, temporal lobe, insula, and along the central sulcus. Entropy values in subcortical regions were higher than those in cortical regions.

### Sensitivity to head motion: windowed method vs. conventional approach

**Figure 6:**
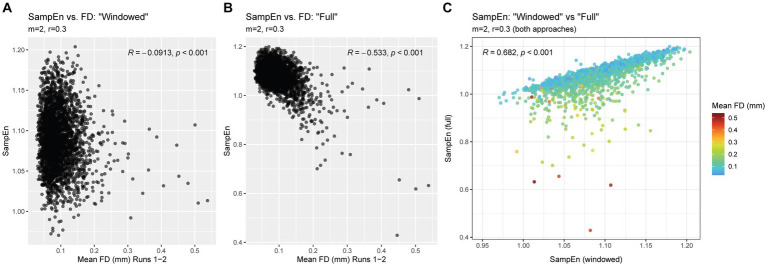
Relationship between SampEn and FD in “full” and “windowed” methods. Panel **(A)** shows the relationship between SampEn values and head motion after using powseR’s windowing approach. Panel **(B)** shows the same relationship, using a conventional approach (where the full, uninterrupted time series is used to calculate SampEn). Panel **(C)** shows the relationship between SampEn values derived from these two approaches, with a color scale applied to indicate the mean FD of each observation.

As expected, the conventional (“full”) approach, using parameters matching those used in the windowed approach (*m* = 2, *r* = 0.3), resulted in SampEn values that were highly correlated with motion. In contrast, SampEn values produced using the windowing approach had a negligible relationship with motion. Scatterplots and correlation coefficients are provided in Figures 6A,B.

Despite the stark differences in relationship with head motion, the windowing approach produced SampEn values that were strongly correlated with results from the traditional “full” approach. Figure 6C illustrates the strong linear correspondence, particularly among participants with low head motion. Participants with higher levels of head motion tended to have markedly lower SampEn in the “full” approach compared to the windowing approach, suggesting that these artifactually lower SampEn values were corrected via powseR’s windowing method.

### Reproducibility of entropy measures in the ABCD study data

**Figure 7:**
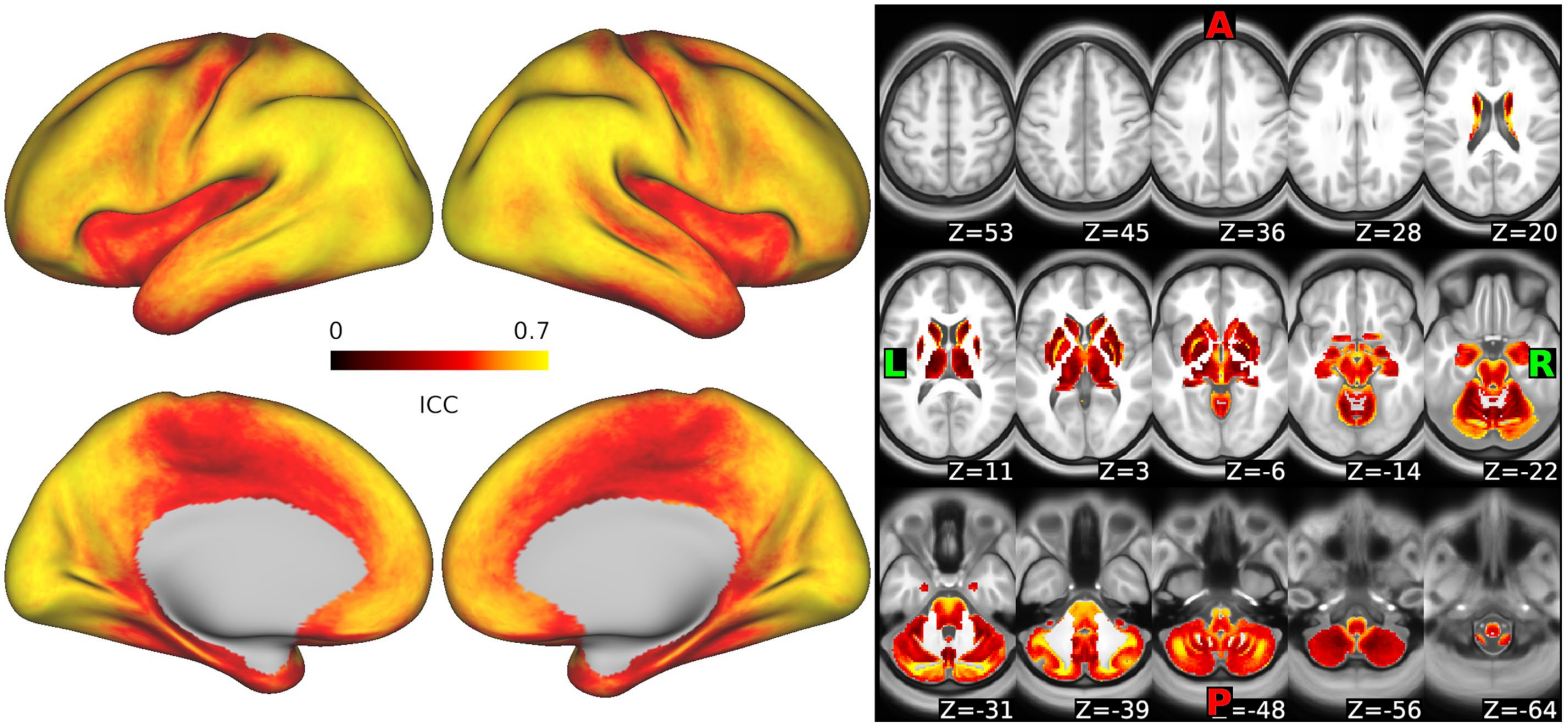
Reliability of sample entropy measurement across the scan. Intraclass correlation values representing the similarity in measurements of sample entropy as calculated from runs 1 + 2 vs. runs 3 + 4 are shown for each vertex in the cortex and each voxel in the subcortex. Warm colors represent high reliability, darker colors reflect lower reliability.

Whole-brain maps of the dense intraclass correlation (ICC) values representing the similarity of SampEn measurements from the data in runs 1 + 2 vs. the data in runs 3 + 4 are shown in Figure 7. ICC values varied across the brain. Median ICCs demonstrated moderately high reliability, though values were somewhat lower in subcortical regions. For cortical surface vertices, the ICC minimum, maximum, median, first, and third quartiles, respectively, were 0.178, 0.735, 0.579, 0.497, and 0.727. For voxels in the subcortex, these values were 0.075, 0.865, 0.421, 0.332, and 0.505, respectively.

## Discussion

We developed a new approach to measuring sample entropy that minimizes the impact of motion and that could be applied across datasets through user-identified adaptations that allow the method to be tailored to the dataset at hand. We compiled these tools into powseR, a publicly available R package and applied them to a large dataset as a demonstration and an opportunity to illuminate some interesting patterns in the whole-brain dense entropy maps that suggest new questions for the field.

The first key takeaway from this work is the association between participant head motion and measures of sample entropy, which likely introduce an unacceptable confound to the analysis if not appropriately addressed. Adding to prior work (9), our results show that SampEn measures correlate with head motion. Notably, these correlations are present even after traditional reprocessing steps designed to remove motion effects (motion correction, motion regression, white matter/CSF/whole brain regression, bandpass filtering) are applied prior to computation of SampEn. While it is critical to extract the best parts of the data (those least influenced by motion) prior to calculating SampEn, it is also a priority to preserve as much naturally-continuous data as possible. This stubborn issue cannot be handled using standard approaches such as traditional scrubbing, because such approaches introduce bias and can worsen motion effects, as both time-series length and number of censored time-points may bias SampEn measures (14, 16, 20). Thus, to avoid confounds related to motion, we adopted a procedure that Dong and colleagues (14) developed to handle missing values in time series data reflecting physiological signals. In this adaptation, we select low motion, continuous excerpts of the time series and combine them to arrive at entropy estimates that are no longer correlated with motion.

The second key takeaway from the work is that we created a tool to allow researchers to automatically determine the optimal *m* parameter, and to use that *m* to test several *r* values to help select the one that best minimizes the relative error. This will advance the field by avoiding the problem of relying on previously-published values for *m* and *r*, and rather allowing researchers to optimize their parameters based on their own data. Identifying the optimal parameters for *m* and *r* may in some cases shed light on the constraints for the reliable application of SampEn methods to fMRI data, which generally has relatively fewer available time points than other kinds of biological signal measurements. For example, if the optimal value for *m* is large, there will be fewer opportunities within the time series for possible matches, so that entropy may not be defined. Also, prior research has shown that time series with shorter lengths tend to require larger *r* values (29) which further highlights the need for caution when using short time series, an issue that becomes exacerbated when all the data cannot be used due to motion confounds.

Third, we created an R package that implements these innovations to allow researchers to (1) identify the best parameters and (2) calculate SampEn using the windowing approach, simultaneously at every vertex and voxel in the brain, creating a whole-brain dense entropy map in CIFTI space. While powseR is focused on SampEn, we envision it becoming just one module in a larger package that will facilitate other promising approaches to analyzing complexity of fMRI data in R, including multiscale entropy, fuzzy, and wavelet-based methods, which would provide a complimentary package in R to the Complexity Toolbox available in MATLAB.^2^

Fourth, the whole-brain dense entropy maps reveal region-specific variation in SampEn values. In similar prior work, we found that subcortical regions had higher entropy values than cortical regions (17, 33). We also found variability across the brain in reproducibility of SampEn measurement, which will be important for researchers in designing studies that would depend on these values (e.g., to conduct brain-behavior analyses.)

While the work presented here includes some key strengths, such as the use of a large, publicly available dataset, the robust consideration of motion effects, and the careful examination of both reliability and change over time in a large sample, it is also important to note several key limitations. First, the windowing approach can only include datasets which contain at least a minimum number of windows (which themselves contain a certain number of continuous “acceptable” time points). Participants who do not have enough total “usable” volumes will necessarily need to be excluded because of the requirement of continuous “usable” volumes in each of the windows. We have found that this limitation is acceptable given the significant benefit of removing/reducing motion-related effects in the entropy calculation. However, this limitation will be particularly relevant to studies with shorter scanning times. Second, our selection of 20 windows of length 20 volumes is somewhat arbitrary and may not be optimal for all datasets. This selection was based on the goal of including enough time points to accurately measure sample entropy while including the maximum number of study participants, given a dataset with substantial head motion contamination. Future work is needed to investigate more quantitative methods for determining what window length and/or number of windows are necessary to achieve accurate and reliable SampEn calculations for a given dataset. Third, in selecting the *m* and *r* parameters, our approach allows the user to find the optimal parameters across participants and across brain grayordinates, but ultimately we have recommended using this information to select a single *m* and *r* parameter to apply across participants and brain regions in creating the whole-brain dense entropy maps. However, it may be that SampEn could be further optimized by allowing individual-specific and/or region-specific parameter selection. This question requires further research. Fourth, there is a need to identify best practices for temporal filtering prior to entropy measurement. For example, prior resting-state fMRI research has shown higher mean frequency signaling in limbic regions (27), suggesting that the impact of different approaches for temporal filtering on region-specific entropy measurement requires additional study. Fifth, the impact of other noise sources, such as respiratory and cardiac, scanner instability, and thermal noise variability between study participants may also affect SampEn. Additional studies investigating how these noise sources impact SampEn values are warranted.

Finally, we acknowledge that there are many different ways to measure the entropy of a brain signal, and SampEn is just one of these methods. We have focused on SampEn because it is both a commonly used method for assessing entropy in BOLD signals but also because of the simplicity of windowing the fMRI data, i.e. For excluding time points with excessive head motion. of approach. However, a potential limitation to using SampEn on such data is that the native sampling rate for the ABCD data (800 ms) is higher than the timescale (0.01–0.08 Hz) usually associated with neuronal contributions to the fMRI signal. For this reason our method may not fully capture the neural information in the fMRI data. Multiscale entropy (MSE) (34), in which the input time series are downsampling the temporal resolution of the time series by averaging adjacent timepoints, is an entropy method that better matches the input temporal resolution of the data to the expected fMRI timescales. A future direction for this approach could be to apply the windowing idea to the MSE approach. The challenge will be that by averaging multiple timepoints together the fraction of MSE timepoints with at least one motion contaminated volume will increase, reducing the number and number of contiguous time points. To our knowledge, the windowing approach we have proposed here has not yet been implemented for multiscale entropy.

In conclusion, we present a suite of tools for optimized calculation of SampEn using automated, data-driven parameter selection and a windowing procedure to minimize the impact of motion while maintaining the natural data structure and avoiding the bias of different time series lengths, that is publicly available in the powseR package. We showed that application of these tools to a large, publicly available dataset reveals reliable SampEn measurement that is resistant to motion-driven distortions. This set of tools has the potential for broad application to advance understanding of brain signal complexity and its role in human development as well as disease processes.

## Data availability statement

Publicly available datasets were analyzed in this study. This data can be found at: https://nda.nih.gov/abcd.

## Ethics statement

The studies involving humans were approved by the University of Minnesota Institutional Review Board. The studies were conducted in accordance with the local legislation and institutional requirements. Written informed consent for participation in this study was provided by the participants’ legal guardians/next of kin.

## Author contributions

DR: Data curation, Formal analysis, Investigation, Methodology, Software, Validation, Visualization, Writing – original draft, Writing – review & editing. JB: Conceptualization, Formal analysis, Investigation, Methodology, Software, Validation, Visualization, Writing – original draft, Writing – review & editing. CF: Conceptualization, Formal analysis, Investigation, Methodology, Software, Writing – original draft, Writing – review & editing. MF: Formal analysis, Investigation, Methodology, Supervision, Validation, Writing – original draft, Writing – review & editing. BK-D: Funding acquisition, Investigation, Project administration, Supervision, Writing – review & editing. BM: Conceptualization, Data curation, Investigation, Methodology, Project administration, Supervision, Writing – review & editing. KC: Conceptualization, Funding acquisition, Investigation, Methodology, Project administration, Resources, Supervision, Writing – original draft, Writing – review & editing.

## Footnotes

^1^https://nda.nih.gov/abcd/

^2^https://www.fil.ion.ucl.ac.uk/spm/ext/#Complexity
